# Beam-Selection for 5G/B5G Networks Using Machine Learning: A Comparative Study

**DOI:** 10.3390/s23062967

**Published:** 2023-03-09

**Authors:** Efstratios Chatzoglou, Sotirios K. Goudos

**Affiliations:** 1 Department of Computer Science, Hellenic Open University, Aristotelous 18, 26335 Patra, Greece; 2 ELEDIA@AUTH, Department of Physics, Aristotle University of Thessaloniki, 54124 Thessaloniki, Greece

**Keywords:** 5G, MIMO, B5G, beam selection, machine learning, V2I, V2X, deep learning, ensemble learning, classification

## Abstract

A challenging problem in millimeter wave (mmWave) communications for the fifth generation of cellular communications and beyond (5G/B5G) is the beam selection problem. This is due to severe attenuation and penetration losses that are inherent in the mmWave band. Thus, the beam selection problem for mmWave links in a vehicular scenario can be solved as an exhaustive search among all candidate beam pairs. However, this approach cannot be assuredly completed within short contact times. On the other hand, machine learning (ML) has the potential to significantly advance 5G/B5G technology, as evidenced by the growing complexity of constructing cellular networks. In this work, we perform a comparative study of using different ML methods to solve the beam selection problem. We use a common dataset for this scenario found in the literature. We increase the accuracy of these results by approximately 30%. Moreover, we extend the given dataset by producing additional synthetic data. We apply ensemble learning techniques and obtain results with about 94% accuracy. The novelty of our work lies in the fact that we improve the existing dataset by adding more synthetic data and by designing a custom ensemble learning method for the problem at hand.

## 1. Introduction

One of the 5G proposals involves using machine learning tools to enhance communications, but this requires a lot of data to test algorithms and assess system performance. In addition, millimeter wave (mmWave) measurements for research in 5G multiple input multiple output (MIMO) require expensive campaigns in elaborated outside areas [1]. Real data related to wave propagation is difficult to obtain because of the time needed to set up the environment and configure the equipment, as well as the cost associated with the purchase of specialized hardware and human resources. This is one of the biggest challenges for 5G data generation, apart from Blockchain [2]. In this situation, synthetic data is a workable substitute as long as the environments in which it is generated are scalable and have a relatively low cost.

The problem of vehicular-to-infrastructure (V2I) or more general vehicle-to-everything (V2X) communication has attracted a lot of attention from the literature in the context of current 5G networks and in future 6G networks [3].

It is well-known that communication in the mmWave band faces several difficulties because of high attenuation and penetration loss. These problems can be overcome by phased arrays with directional beamforming by concentrating RF energy at the receiver [4,5].

In vehicular systems, mmWave MIMO is a method of exchanging sensor data, according to [6]. The fact that mmWave, as originally intended for this application, necessitates the pointing of narrow beams at both the transmitter and receiver poses a significant challenge. One of the interesting problems in this context consists of selecting the ideal pair of beams for analog beamforming (BF), with fixed beam codebooks and antenna arrays on both the transmitter and the receiver [7]. The authors of [8] provide a comprehensive overview of ML techniques for mmWave BF deployment in future wireless networks.

The research in [7] provides a dataset of small-scale parameters obtained from ray-tracing (RT) simulations, in which the simulation’s time-related snapshots resemble video frames. In this work, we apply several ML methods to produce better results than those found in the literature. More specifically, we apply both shallow and deep learners. We produce additional synthetic data (oversample) with Synthetic Minority Over-sampling Technique (SMOTE) [9] and we fine-tune the ML models using the GridSearch algorithm. The obtained results outperform those from the literature.

The main contributions of this work are summarized as follows:

- We provide an effective way to produce synthetic data for a common dataset found in the literature.
- We propose an ensemble learning composed of base shallow learners method to obtain more accurate results with low complexity.
- We show that an optimal beam pair can be obtained using our approach up to an accuracy of about 94%.

To the best of the authors’ knowledge, this is the first time that an ensemble of classifiers is applied to the specific problem for optimal beam pair selection in 5G/6G cellular communications. The rest of this paper is organized as follows. Section 2 presents the related work. The problem description and the preprocessing process are given in Section 3. The numerical results are presented in Section 5. Finally, we discuss the results in Section 6, while the conclusion is drawn in Section 7.

## 2. Related Work

There are several survey papers in the literature that discuss different aspects of ML application to future wireless networks. The topic of antenna design for future networks using ML is discussed by the authors in [10]. In contrast, the topic of ML applications in vehicular networks from the networking point of view is examined in [11]. In the same context, the authors provide a thorough overview of various machine learning techniques applied to communication, network, and security components in a vehicular network in the works [12,13]. In another recent study, the authors in [14] report the most recent developments in the area of deep learning-based physical layer methods to pave the way for emerging applications of 6G.

Our contribution focused on the RAYMOBTIME dataset [7]. The latter dataset contains different simulated scenarios with Simulation of Urban MObility (SUMO) [15], i.e., a simulated automotive traffic program. The current dataset has already been analyzed by the literature, due to the diversity of options it offers. However, simpler studies are quite absent, i.e., most studies use extensive machine learning models to achieve optimal results.

First, in 2019, the authors of [16] analyzed this dataset with Deep Neural Networks (DNNs). With the assistance of LIDAR and separating their analysis into four different machine learning models, they managed to obtain 91.2% accuracy. The LIDAR sensor is responsible for identifying traces of light in order to calculate the distance between two objects.

Next, in 2021, the authors in [17] focused again on LIDAR technology, i.e., they used a Federated Learning methodology with a Convolutional Neural Network (CNN) of six layers, ReLU and PReLU activators, and the Softmax as the output function.

After that, the authors in [18] used both supervised and unsupervised ML models in their analysis. In the case of unsupervised learning, the mean absolute error (MAE) was 6.15.

In 2022, the authors in [19] propose a beam selection model, that will be based on CNN. Specifically, the latter CNN model should contain six layers (2D) and a linear layer. The GPS data were used at this point, and then four more linear layers were added. All layers had the ReLU activator, except the last one, which used Softmax. Their results showed an accuracy of 96.9% for the top-10 accuracy value for both the S008 and S009 sets.

The following study cited in [5] used both GPS and LIDAR data to improve the prediction rate of beam selection on top-10 accuracy. This analysis managed to achieve a top-10 accuracy of 91.11% with S008.

Authors in [20] proposed a backdoor attack algorithm to exploit the Federated Learning (FL) based mmWave beam selection method. Precisely, their attack triggered obstacles at specific locations on the road. They designed two target tasks; (a) to force the model to output a beam in a desired direction after the attack was triggered, and (b) to cause the model to send a beam with low signal strength. After confirming their experiments, they say that the beam selection system based on FL does not completely protect user privacy against certain attacks. To this end, they proposed a new defense method that can invalidate backdoor attacks, i.e., a dynamic norm clipping method that dynamically adjusts the size of the server clips, along with a backdoor detection method to efficiently provide a reliable signal. In the second case, a federated noise titration algorithm was proposed to show how the global model reacts to inputs with different levels of noise.

In [5], the authors designed deep learning architectures that can predict a set of top-K beam pairs with the assistance of non-RF sensor data such as GPS, camera, and LiDAR, improving the prediction accuracy by 3.32 to 43.9%. They also proposed that a fusion network exhibits 20–22% improvement in top-10 accuracy when comparing their technique against other state-of-the-art ones. Additionally, they calculated an optimization problem to choose the set of K candidate beam pairs, which decreased the beam selection time by 95–96%.

The authors of [21] proposed an estimation approach for both conventional and Reconfigurable Intelligent Surfaces (RIS) assisted massive MIMO systems of FL-based models. Input and output data were combined for each communication link, instead of using different neural networks for each task. They also proved the convergence of FL and demonstrated its superior performance over centralized learning (CL).

The authors propose FusionNet in [22], a dual-input neural network to predict the optimal beam using both the sub-6 GHz channels. They also used a deep learning method to activate the mmWave band antennas. The FusionNet outperformed the traditional fully connected neural network and the existing sub-6 GHz strategies in terms of both prediction accuracy and achievable rate.

However, in none of the above studies did the authors generate synthetic data to increase the dataset size. Additionally, we have not been able to find a study in the literature that used an ensemble learning approach for this dataset. Therefore, the goals of this study are to both augment the current dataset and improve accuracy results by introducing ensemble learning techniques.

## 3. Preliminaries

The authors in [7] have produced the dataset by combining simulations from ray tracing (RT) commercial software with the Simulation of Urban MObility (SUMO) traffic simulator. The dataset is generated representing a location in Rosslyn, VA, USA. Beam-selection is configured as a classification task with the optimal beam pair index as the target output. The operation frequency was 60 GHz.

In this study, we use the S000 scenario, the sane one the authors had analyzed in their study [7]. Regarding the S000 scenario and the data preprocessing steps, we used the ones the same authors had implemented in their study [7], i.e., they are directly related to the beam selection problem and the choice the antenna has to make during the beamforming process. This means that for the sake of Quality-of-Service (QoS), the antenna has to predict the next position of the user and send the current beam in the proper direction, eventually improving the whole QoS experience for each user.

## 4. Performance Metrics

In the remainder of this paper, we use the following multiclass classification metrics: [23,24].

1. *Accuracy* is defined as the fraction of correct predictions. It is expressed as
  (1) $$Accuracy=\frac{1}{N}\sum_{n=1}^{N-1}1\left({\hat{z}}_{n}=z_{n}\right)$$
  where *N* is the number of test classes, ${\hat{z}}_{n}$ denotes the ML model class predicted label, $z_{n}$ is the true class label, and 1(x) is the indicator function.
2. *Top-k Accuracy* can be considered as a generalization of accuracy. The differentiation is that a prediction is regarded as accurate if the true label is connected to one of the top k forecasted scores. This is defined mathematically as
  (2) $$\mathrm{Top}-k\mathrm{accuracy}\left(z,\hat{f}\right)=\frac{1}{N}\sum_{n=0}^{N-1}\sum_{m=1}^{k}1\left({\hat{f}}_{n,m}=z_{n}\right)$$
  where ${\hat{f}}_{n,m}$ denotes the predicted class for the *n*-th sample that corresponds to the *m*-th largest predicted score.
3. *Precision*, which in multiclass classification is defined as
  (3) $$Precision=\frac{\sum_{c=1}^{C}TP_{c}}{\sum_{c=1}^{C}TP_{c}+FP_{c}}$$
  where *C* is the number of classes, $TP_{c}$ is the number of true positive, and $FP_{c}$ is the number of false positive for class *c*, respectively.
4. *Recall* is expressed as
  (4) $$Recall=\frac{\sum_{c=1}^{C}TP_{c}}{\sum_{c=1}^{C}TP_{c}+FN_{c}}$$
  where *C* is the number of classes, $TP_{c}$ is the number of true positive, and $FN_{c}$ is the number of false negative for class *c* respectively.
5. *F1* which aggregates Precision and Recall under the concept of harmonic mean. This is defined as
  (5) $$F1=2\times \frac{\mathrm{Precision}\times \mathrm{Recall}}{\mathrm{Precision}+\mathrm{Recall}}.$$
  *F1* can be considered as a weighted average between Precision and Recall.
6. *Area Under the Curve* (AUC) is the measure of a classifier’s ability to differentiate between classes. AUC computes the area under the receiver operating characteristic (ROC) curve. This summarizes the curve information into a single number. ROC is a probability curve, whereas AUC is a measure or degree of separability. It indicates how well the model can differentiate between classes. The model performs better with a higher AUC. In multiclass classification problems, the One-vs-one algorithm is a common solution. This algorithm determines the average AUC for all possible pairwise class combinations. The definition of this multiclass AUC metric, which is weighted uniformly, is given mathematically by [25]
  (6) $$\frac{1}{C(C-1)}\sum_{i=1}^{C}\sum_{m>i}^{C}(\mathrm{AUC}\left(i|m\right)+\mathrm{AUC}\left(m|i\right)$$
  where AUC(i|m) defines the AUC with class *i* as the positive class and class *m* as the negative class. In general, it is AUC(i|m)≠AUC(m|i)) in the multiclass case.

For Regression we will use the metric Mean Absolute Error (MAE), which is defined as [26]:

(7) $$MAE=\frac{1}{n}\sum_{i=1}^{n}\left|y_{i}-{\bar{y}}_{i}\right|$$

where *n* denotes the number of test patterns, *y* are the real measured data and $\bar{y}$ the predicted ones of the *i*-th data record.

## 5. Numerical Results

The main dataset was the same as in [7] in order to compare results. To identify the best ML model, we analyzed both shallow and Deep learning models. The reason is that shallow ML models can achieve good results faster and without losing much time configuring each model, but they cannot solve very complex problems. On the other hand, deep learning models can solve complex problems, but they need proper time to experiment and identify the optimal Deep learning network that we can create, i.e., the number of nodes, the number of layers, the correct choice of optimizer for each layer, etc. [27]. Our analysis followed the steps below:

1. Initial shallow analysis using several different ML learners, using the ML models of the original work [7].
2. Data augmentation with SMOTEN, to increase the samples of each class to at least 10. After that, we could analyze the dataset with the 10k stratified CV method.
3. Optimal hyperparameters search using the GridSearch algorithm.
4. Deep Learning analysis with both classification and regression tasks.

The numerical results were run on a Windows 10 Pro machine with an AMD Ryzen 7 2700, 64 GB of RAM, and a GTX 1060 6 GB GPU. Note that only the DNN models used the GPU as processing power; the shallow analysis was made with the CPU. The Python language with common libraries was used for the execution of ML models. Specifically, we used sklearn and imbalanced-learn.

### 5.1. Shallow Learning

Ensemble learning methods are a popular framework for machine learning applications, according to [28]. These methods train combinations of base models or base learners, which can be decision trees, neural networks, or any other supervised learning technique. Both regression and classification problems have been solved using ensemble methods. These methods have gained popularity because they have outperformed other single-model methods in multiple instances. The base learners to be combined in ensemble learning may be of the same or different types.

As a combination technique, learners like Random Forest (RF) [29] use Bootstrap Aggregating (or Bagging) [30]. This method improves the performance of individual trees by employing a voting scheme between their predictions. Prior to voting, each tree is grown using a distinct subset of the available training data to achieve diversity among the trees and reap the benefits of averaging the predictions of the uniquely grown trees. On the other hand, a Voting Classifier trains multiple models using different selected algorithms and returns the classification result based on the majority vote.

Ensemble models are valuable options, and they regularly produce excellent outcomes. They are less likely to overfit the data because they train multiple models with different data subsets. Since there are more models confirming the classification is in the right direction, they can provide greater precision.

First, we made a basic comparison with the initial results of the authors of RAYMOBTIME presented in [7]. We apply the following learners Linear Support Vector Classifier (LinearSVC) [31], Adaptive Boosting (AdaBoost) [32], Decision Trees (DT) [33], Random Forest (RF) [29], Deep Neural Network (DNN) [34], Naive Bayes [35], Artificial Neural Network (ANN) [36], k-Nearest Neighbors (kNN) [37], Stochastic Gradient Descent (SGD) Classifier [38], light gradient-boosting machine (LightGBM) [39], Logistic Regression [31], Extra Trees (ET) [40]. Table 1 presents the results of our analysis. As can be observed, there are small differences (2 to 5%) between the past and the current analysis. Even with other classification ML models, like Extra Trees, the results are not very accurate. The results indicate that our analysis is correct and very close to that of [7], as presented in the same table.

After further examination of the provided samples, it was observed that five classes had only one sample, and while the dataset was split into training and testing sets, these two sets did not contain the same number of samples. For example, a class had only one sample, and it was only on the training set. This made the evaluation phase of each ML model hard to predict because the models had to predict samples they did not know. For this reason, we removed these five classes (5, 10, 22, 38, and 54), merge the training and testing sets into one dataset, and split it again with a 70/30% analogy. It should be noted that by removing these five classes, the number of classes and the number of samples were reduced by 10% and by 0.01%, respectively. Since the analysis is based on an uneven set of data, we will focus on the AUC score to figure out how well we can predict. However, for the sake of completeness, the accuracy, and top-k metrics will also be present if they are applicable. We executed a new analysis with the same ML algorithms, but with 56 classes, as shown in Table 2. As can be observed, almost all ML models surpass the 50% threshold of accuracy by removing these classes. Specifically, only Naive Bayes presented worse results, in comparison to the 61 classes of analysis. About 90% of the ML models presented better results with 56 classes. On top of that, the results were improved by 40% in some cases, such as on DT.

### 5.2. Data Augmentation

To further improve the results of each classification analysis, a stratified k-fold cross-validation analysis should be made. To achieve this, each class must have at least 10 samples, to be able for the dataset to be split into 10 test sets. A dataset is considered to be imbalanced if the classes are not approximately equally represented. Thus, the specific dataset is imbalanced and we need to apply a data augmentation algorithm to correct this problem. For this reason, we acquired the SMOTE technique to oversample the dataset. This technique can create new samples based on the classes, by implementing the kNN algorithm.

Specifically, we used the Synthetic Minority Over-sampling TEchnique-Nominal Continuous (SMOTE-NC) algorithm [9], which is the SMOTE variant for oversampling mixed datasets of continuous and nominal features, with k_neighbors equal to 3 and random_state equals to 42. Since the k_neighbors parameter was equal to 3, each class should have at least 3 samples. So, to avoid overfitting the dataset with unnecessary data, we used the following pseudocode code of listing in Algorithm 1.
**Algorithm 1** Pseudocode for SMOTE-NC samples separation.1:**while** i<numOfFeatures **do**2:   calculate sum of unique values per feature3:   **if** sum<26 **then**4:     **if** sum<4 **then**5:        remove this feature from the dataset6:        populate the values of this feature, to be more than 37:        add this feature into a new subset8:        continue9:       **end if**10:     remove this feature from the dataset11:     add this feature into a new subset12:   **end if**13:**end while**

Specifically, the Algorithm 1 kept one specific class, as the majority one, in order for the remaining classes to reach this number of samples. Then, if a class had fewer than four samples, their samples were re-copied. For instance, if a class had two samples, it got four samples, which were the same. With this method, all classes had at least three samples, for the SMOTE-NC algorithm to be able to oversample the dataset. After the Algorithm 1 execution, the SMOTE-NC algorithm was executed only to the training set. As a result, an additional 1311 samples (0.04%) were added to 28 classes, increasing the original sample size of the training set from 28,712 to 29,858.

### 5.3. Optimal Hyperparameters Search

In order to obtain the best performance we need to find the optimal hyperparameters for each shallow algorithm that we mentioned in Section 5.1. First, we made an analysis with GridSearchCV, to identify the latter hyperparameters. Second, we analyzed the shallow algorithms with the hyperparameters that GridSearchCV found.

#### 5.3.1. GridSearchCV

To find the optimal hyperparameters, each shallow algorithm was executed with GridSearchCV 10 times. By using the Data Augmentation technique in Section 5.2, we managed to have at least 10 samples in each class of the dataset. Before executing GridSearchCV, we merged the SMOTE-NC training set and the test set into one dataset. As a result, each training set had 37,947 samples and each test set had 4217 samples, for each of the CV splits. To avoid overfitting, we executed the SMOTE-NC algorithm before executing GridSearchCV. This means that each ML model examined different oversampling data.

Table 3 illustrates the results of each algorithm. We were able to improve the prediction rate in most cases by fine-tuning each ML algorithm. Especially with LightGBM, which increased the prediction rate of the AUC score by ≈45%, reaching the highest AUC score so far, i.e., 85.38%.

#### 5.3.2. Stratified Cross-Validation Results

After completing the analysis of Section 5.2 and Section 5.3, a question remains: can these models achieve the same results with stratified 10-fold cross-validation? And what will be the prediction rate of more complicated ML models, such as ensemble learning models? To this end, we added four ensemble methods, i.e., Voting-4, Voting-8, Bagging-DT, and Bagging-RF. Voting-4 contains the four best ML models, based on their AUC score, i.e., DT, NN, kNN, and LightGBM, while Voting-8 contains all ML models except LinearSVC, SGDClassifier, and Logistic Regression. Both voting algorithms used the “soft” method to predict the testing set. In soft voting, each individual classifier provides a probability that a particular data point belongs to a specific target class. The predictions are summed and weighted according to the importance of the classifier. The winner is the target label with the highest sum of weighted probabilities. This study analyzes and tries to improve the accuracy of the beamforming selection, with the purpose of increasing the quality of service for each user. Because of this, even a small improvement in beamforming selection can be seen as a success. Soft voting allows the selection of the beam pair with the highest probability. For this reason, we added the top-k accuracy metric and used soft voting.

Additionally, Bagging-DT uses the DT as the base estimator, while the Bagging-RF model uses the RF. Both Bagging models use the following parameters: bootstrap is false, the number of max_samples is equal to 10,000, the number of n_estimators is 100, and the oob_score is false.

Based on these parameters, Table 4 contains the results of all algorithms along with the ensemble models. Note that this table also contains the remaining evaluation metrics, such as the Precision (Prec), Recall, and F1 score. As can be observed from this table, the results in the AUC score are improving by 10% in each algorithm. This means that the process we used worked to make each ML model better at making predictions. Only Logistic Regression showed a smaller increase in the prediction rate, i.e., 6%, which was based on the convergence of this algorithm. This means that this algorithm needed more iterations to be trained correctly, and as a result, did not obtain the optimal training phase. Finally, 8 of the 15 ML models presented had an AUC score of more than 90%, which is a significant improvement over the results in Table 1. Voting-4 had the best AUC score with 94.5%.

### 5.4. DNN Analysis

We performed both classification and regression analysis using DL models. For the former analysis, two different DNN models were used, i.e., Multi-layer Perceptrons (MLP) and Denoising Stacked Autoencoders. For the latter analysis, only the CNN model was tested. Each analysis was implemented with a stratified 10-fold cross-validation. To achieve this, the SMOTE-NC algorithm was used, as described in Section 5.2.

#### 5.4.1. DNN Classification

Each DNN model uses Model Checkpoint and Early Stopping. The former is capable of saving the weights after each epoch, while the latter is responsible for stopping the training phase if a targeted metric stop improves. In this study, the latter metric is loss. If the loss metric does not improve for two consecutive epochs, the training phase is terminated and the DNN model is re-trained using the most recent model checkpoint weights (optimal state). Table 5 highlights the details of each DNN model.

Table 6 highlights the results of each DNN model. As can be seen, DNN models were not able to predict more accurately than the Voting-4 model. However, both results, especially the AUC score of MLP are quite high (93.07%). So, these results validate that the methodology followed can improve the prediction rate of any ML model.

**Figure 1:**
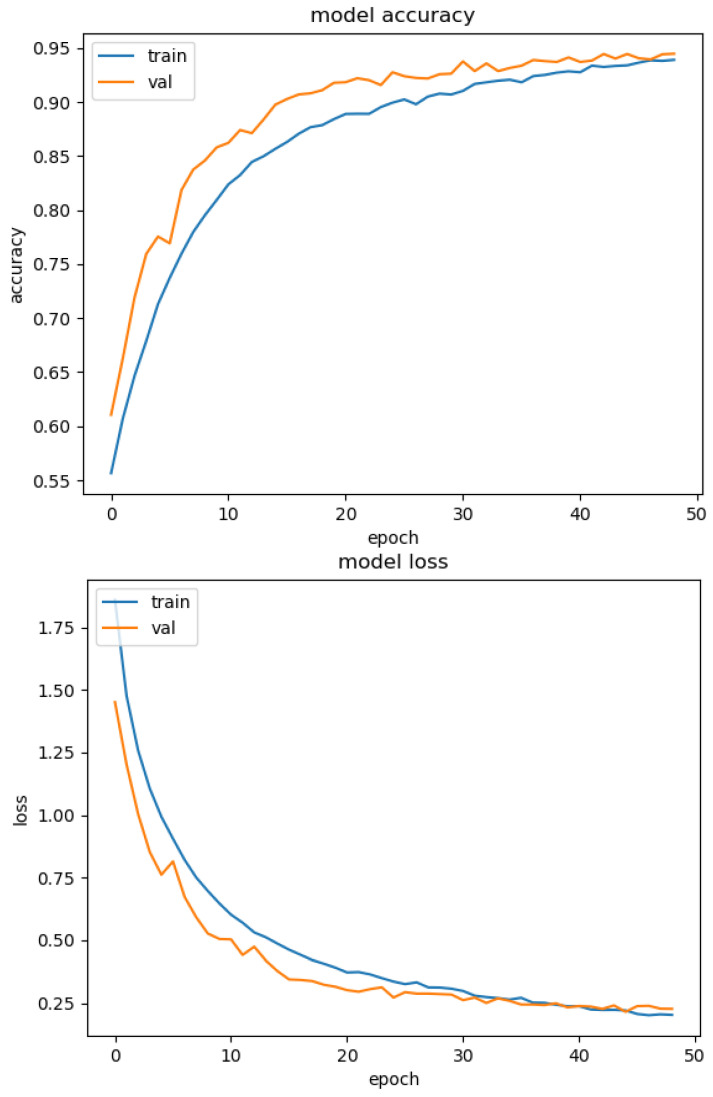
MLP accuracy per epoch (**top**) and lost per epoch (**bottom**) for the 1st fold training phase.

**Figure 2:**
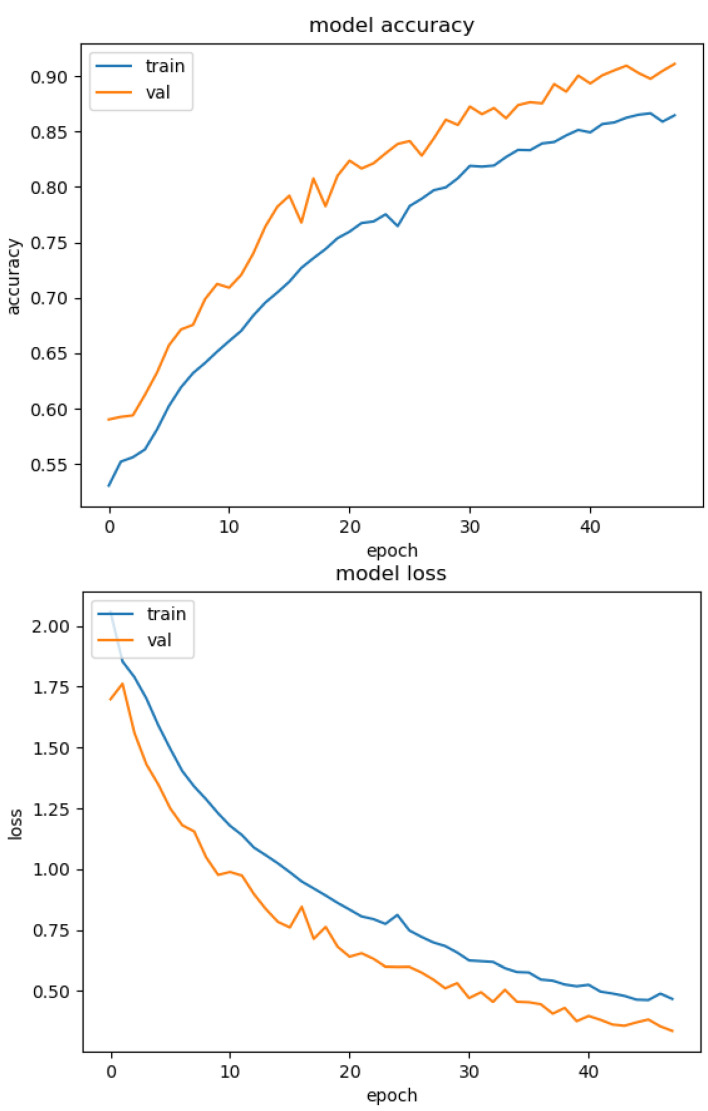
Autoencoder accuracy per epoch (**top**) and lost per epoch (**bottom**) for the 1st fold training phase.

Figure 1 and Figure 2 illustrate the graph of the accuracy and the lost metrics per epoch for the 1st fold cross-validation training phase. This shows that the DNN models avoid overfitting, by using the Model Checkpoint, Early Stopping, and Dropout techniques. For instance, in the case of Figure 1 after the 40th epoch, the training accuracy is the same as or lower than the validation accuracy. So, the model stopped the training phase to avoid overfitting and retrained back to the optimal weight values.

#### 5.4.2. DNN Regression

Regarding the regression analysis, we first examined the provided model of the authors, as stated in the GitHub repository [41]. So, we analyzed this model with the authors’ dataset for 500 epochs. The result was a Mean Square Percentage Error (MAPE) score equal to 85.34%, which is quite high.

For this reason, we created a new CNN model that was analyzed with the methodology of Section 5.1 and Section 5.2. This CNN ML model had the input Keras layer function, which was assigned the value of the total columns (5750). Then, the model had three consecutive layers, (a) a Conv1D, (b) an AveragePooling1D, and (c) a Batch normalization layer. After these layers, the activation function was placed, i.e., ReLU. These layers were added three times, and then a Dense layer was placed with 100 nodes. After that, a Dropout layer of 0.8 value was added, leading to the Flatten layer. The last layer had a Dense layer with only one node since the output activator was the Linear function.

As with the DNN classification models of Section 5.4.1, the CNN had the same values for learning rate and momentum. Also, CNN used the same optimizer, i.e., Stochastic Gradient Descent (SGD). Additionally, both Model Checkpoint and Early Stopping techniques were implemented, following a similar methodology as with the DNN classification models. Table 7 demonstrates the parameters of the CNN ML model.

After analyzing CNN with stratified 10-fold cross-validation with 56 classes and adding the oversampling effect with SMOTE-NC, the average MAE was equal to 5.32, with only 5 epochs needed for the training phase.

## 6. Discussion

The findings of our work suggest that it is quite possible to find the optimal beam pair in 5G networks with a quite high probability of 94%. Such a result is feasible with low complexity without using time-consuming DL methods but rather using an ensemble approach with four base learners.

On the other hand, our work has certain limitations. More specifically, three facts limit our work. First, the low number of samples, or training data, may cause problems during the deep learning training phase. Second, the dataset is synthetic. As a result, as with any synthetic dataset, our ML models may be skewed in a real-world scenario. Third, the dataset needs much data preprocessing work to convert it to a proper set that is going to be analyzed by an ML model.

## 7. Conclusions

We have presented a comparative study of ML methods for the beam selection problem in 5G/B5G networks. We have given a complete framework of actions to improve classification accuracy. The accuracy of our results rises to above 92% using different ML models as well as the SMOTE-NC algorithm for oversampling the imbalanced classes. This is about 30% higher than that of the original paper. Moreover, both DL models gave results with accuracy higher than 90%. However, the higher accuracy values were obtained using ensemble learning techniques, which was expected since these methods could produce better results than individual learners. Both voting classifiers obtained results with accuracy higher than 95%. In addition, our study produced better results without using GPS or LIDAR data. Such data could possibly further improve the accuracy of the beamforming prediction. Also, we mostly used common shallow and Deep Learning models, which can be further improved into more complicated ML models, with the purpose of increasing the accuracy of each ML model. Thus, this study could be improved in future work by using more advanced Deep Learning models and analyzing additional data, like GPS or LIDAR

## Figures and Tables

**Table 1:** Results comparison with the original work of [7], based on the script’s execution that the original authors published with [7]. With bold font are highlighted the optimal results of each column.

Classifiers	Accuracy (%) in [7]	Accuracy (%) This Study
LinearSVC	33.20	35.62
AdaBoost	55.00	54.96
Decision Trees	55.50	52.06
Random Forest	**63.20**	57.88
DNN	**63.20**	**62.20**
Naive Bayes	–	8.70
Artificial Neural Net (ANN)	–	40.50
kNN	–	43.04
SGDClassifier	–	37.80
LightGBM	–	26.66
Logistic Regression	–	43.76
Extra Trees (ET)	–	52.66

**Table 2:** Results based on the execution of each algorithm, but with 56 classes. With bold fold highlighted the optimal results of each column are.

Classifiers	AUC (%)	Accuracy (%)	Top-2	Top-5	Top-10
Naive Bayes	64.18	4.79	10.44	23.11	29.44
Decision Trees	**84.85**	**92.94**	94.39	95.16	95.60
Random Forest	74.45	87.44	96.09	99.16	99.33
AdaBoost	50.20	60.28	70.81	86.36	93.87
LinearSVC	74.42	79.25	–	–	–
NN	67.00	88.96	95.89	98.64	99.18
kNN	76.73	87.68	94.44	97.37	97.47
SGDClassifier	70.46	59.52	–	–	–
LightGBM	49.97	40.49	51.28	51.52	52.40
Logistic Regression	63.49	76.83	90.73	97.23	98.58
ET	50.00	58.52	69.23	85.90	93.53

**Table 3:** Results with the GridSearchCV parameters analysis. Bold highlights best results.

Classifiers	AUC	Accuracy	Top-2	Top-5	Top-10
DT	83.67	93.59	94.62	94.64	94.75
Random Forest	66.49	86.96	96.34	99.48	99.73
NN	84.12	92.35	94.44	99.04	99.48
kNN	76.73	87.68	94.44	97.37	97.47
Logistic Regression	80.16	85.37	95.83	99.21	99.57
Naive Bayes	79.14	14.12	30.97	66.20	84.15
AdaBoost	50.20	60.28	70.78	86.35	93.87
LinearSVC	76.53	83.61	–	–	–
SGDClassifier	72.63	82.86	–	–	–
LightGBM	**85.38**	**95.10**	98.37	99.52	99.71
ET	68.74	85.95	96.22	99.17	99.69

**Table 4:** Stratified 10-fold cross-validation results. Bold highlights best results.

Classifier	AUC	Accuracy	Prec	Recall	F1	Top-2	Top-5	Top-10
DT	93.77	94.56	89.00	87.69	87.54	95.37	95.41	95.54
RF	85.22	87.91	83.67	70.89	74.57	96.63	99.52	99.80
NN	93.15	93.67	89.60	86.49	87.00	97.89	99.41	99.64
kNN	90.66	91.71	90.07	81.57	84.42	90.66	96.39	98.32
Logistic Regression	91.39	85.87	90.25	83.24	85.58	96.19	99.33	99.69
Naive Bayes	79.64	17.22	40.88	71.38	44.74	36.88	68.33	84.95
AdaBoost	50.74	58.71	3.42	3.18	2.55	68.99	84.35	91.84
LinearSVC	89.55	84.48	9.83	79.64	83.12	–	–	–
SGDClassifier	87.72	83.31	85.11	76.01	78.16	–	–	–
LightGBM	93.90	95.64	92.08	87.93	89.18	98.61	99.53	99.77
ET	83.41	85.85	82.46	67.33	71.54	95.98	99.24	99.74
Voting-4	**94.50**	**95.86**	91.89	87.13	89.66	98.79	99.58	99.76
Voting-8	94.12	95.40	92.10	88.39	89.47	98.22	99.59	99.87
Bagging-DT	92.06	94.00	91.57	84.32	86.78	98.74	99.60	99.68
Bagging-RF	87.07	89.55	87.30	74.54	78.54	97.93	99.64	99.81

**Table 5:** DNN classification parameters per model.

Parameters	MLP	Autoencoder
Activator	ReLU	ReLU
Output activator	Softmax	Softmax
Initializer	He_uniform	–
Optimizer	SGD	SGD
Momentum	0.9	0.9
Dropout	0.15/0.1	0.1
Learning rate	0.01	0.01
Loss	SCC	SCC
Batch normalization	Yes	Yes
Layers	11	16
Nodes (per layer)	500/450/400/ 350/300/250/ 200/160/120/ 60/56	300/250/200/ 100/100/50/25/ 10/25/50/100/ 100/200/250/300/ 56
Batch size	32	32

**Table 6:** DNN classification results per model. Bold highlights best results.

Model	AUC	Accuracy	Precision	Recall	F1	Epochs
MLP	**93.07**	**94.18**	85.90	86.29	85.17	50.6
Autoencoder	87.28	91.68	74.78	74.79	73.27	62.5

**Table 7:** DNN regression model parameters.

CNN	
Activator	ReLU
Output activator	Linear
Optimizer	SGD
Momentum	0.9
Dropout	0.8
Learning rate	0.01
Loss	MAE
Batch normalization	Yes
Layers	6
Nodes (per layer)	Conv1D(128,10)/ Conv1D(64,12)/ Conv1D(32,10)/ Dense(100)/ Flatten()/ Dense(1)
Batch size	32

## Data Availability

All data are available within the manuscript.

## Abbreviations

The following abbreviations are used in this manuscript:

CNN	Convolutional Neural Network
DNN	Deep Learning Neural Networks
DT	Decision Trees
ET	Extra Trees
lr	learning_rate
MAE	Mean Absolute Error
ML	Machine Learning
mln	max_leaf_nodes
MLP	Multi-layer Perceptrons
MAPE	Mean Square Percentage Error
msl	min_samples_leaf
mss	min_samples_split
NN	Neural Net
QoS	Quality of Service
ReLU	Rectified Linear Unit
RF	Random Forest
SCC	Sparse Categorical Crossentropy
SGD	Stohastic Gradient Descent
vs	var_smoothing
